# Case Report: A successful percutaneous revascularization of bilateral acute limb ischemia caused by an aortic saddle embolism in a patient with mitral stenosis

**DOI:** 10.3389/fcvm.2026.1797744

**Published:** 2026-07-15

**Authors:** Muhamad Sofan Dhani, Sulistiyati Bayu Utami, Safir Safir, Ilham Uddin

**Affiliations:** 1 Department of Cardiology and Vascular Medicine, Faculty of Medicine, Universitas Diponegoro, Semarang, Indonesia; 2 Diponegoro National Hospital, Semarang, Indonesia; 3 Dr. Kariadi General Hospital, Semarang, Indonesia

**Keywords:** aortic saddle embolism, bilateral acute limb ischemia, mitral stenosis, percutaneous revascularization, percutaneous thromboaspiration

## Abstract

**Background:**

An aortic saddle embolism (ASE) is an uncommon condition involving sudden aortoiliac occlusion where emboli lodge at the aortic bifurcation, causing bilateral lower-extremity arterial occlusion. This can lead to ischemia, potential amputation, or even death due to severe reperfusion injury and multiple organ failure. Prompt and effective restoration of arterial flow is crucial to prevent irreversible tissue infarction and to preserve limb viability. Various revascularization techniques, including endovascular and surgical thrombectomy, can be employed to manage this condition.

**Case illustration:**

A 40-year-old woman presented with severe pain and numbness in both legs and a bluish tinge in both toes. An electrocardiogram showed atrial fibrillation, and transthoracic echocardiography revealed severe mitral stenosis. The patient underwent percutaneous angiography, which revealed total occlusion at the distal abdominal aorta, followed by percutaneous mechanical thrombectomy or percutaneous thromboaspiration, for which a vacuum-assisted device with conventional wall suction was used, which restored flow to the left lower extremity. Subsequently, the patient received heparin therapy, pain management, and anti-inflammatory treatment. A second percutaneous thromboaspiration with additional balloon angioplasty was performed several days later, which successfully restored flow to the right distal extremity. A duplex ultrasound evaluation after the second percutaneous procedure showed normal arterial and venous blood flow in both lower extremities. The patient experienced no significant complications and recovered fully. One year later, the patient underwent mitral valve replacement with good outcomes and no complications.

**Conclusion:**

A modified wall-suction thromboaspiration may represent a feasible bailout or resource-adapted option for bilateral acute limb ischemia caused by an aortic saddle embolism when dedicated thrombectomy devices are unavailable, but the safety, blood loss risk, and reproducibility of this procedure require further evaluation.

## Introduction

An aortic saddle embolism (ASE) is a rare but serious medical condition characterized by the sudden blockage of the aortoiliac arteries, specifically at the point where the abdominal aorta divides into the two iliac arteries (1). In this condition, emboli–blood clots or other debris–become lodged at the aortic bifurcation, resulting in the obstruction of blood flow to both lower limbs simultaneously (2).

This bilateral arterial occlusion can cause critical ischemia, depriving the tissues of oxygen and nutrients, which if left untreated, may rapidly progress to tissue death. The consequences of ASE are severe and can result in limb amputation from irreversible damage and life-threatening complications such as reperfusion injury following restoration of blood flow and multiple organ failure stemming from systemic inflammatory responses. Because of these potentially devastating outcomes, it is essential to quickly and effectively re-establish arterial circulation to prevent permanent tissue infarction and to maximize the chances of saving the affected limbs (3, 4).

Management of ASE involves a range of revascularization strategies, which may include minimally invasive endovascular procedures or open surgical thrombectomy, tailored to the patient's condition and the extent of the embolic obstruction. Early diagnosis and prompt intervention are critical to improving patient prognosis and reducing morbidity and mortality associated with this vascular emergency (5).

In this study, we report the case of a 40-year-old woman who presented with bilateral acute limb ischemia (ALI) caused by an ASE. The patient also had atrial fibrillation (AF) with a previously undiagnosed mitral stenosis (MS) and was treated with vacuum-assisted thromboaspiration using a conventional wall-suction device.

## Case illustration

A 40-year-old woman, who worked as a teacher, presented to the emergency department of Dr. Kariadi General Hospital with severe resting pain in both legs that started 7.5 h prior to admission. She also reported numbness and tingling in her toes, which did not resolve with rest. A physical examination revealed that both legs appeared pale with mottled discoloration on the soles, felt cold to the touch, a lack of pulse at both lower extremities, and unmeasurable peripheral saturation. However, she could still move both her legs (Table 1). She did not have any previous medical history and was not taking any medications at home.

**Table 1:** The limb signs and status of Rutherford IIa taken from a bedside physical and Doppler examination in this patient with acute limb ischemia (ALI).

Rutherford grade	Category	Sensory loss	Motor deficit	Capillary refill of lower extremities	Arterial Doppler signal	Venous Doppler signal	Description
IIa	Marginally Threatened	Minimal (Partial). Numbness and tingling in the toes	None	Slow intact (>2 s/ > 2 s)	Inaudible	Audible	Salvageable if promptly treated

In the physical examination, cardiac auscultation revealed a grade II/4 low-pitched, mid-diastolic rumbling murmur at the apex with left lateral decubitus maneuver. There was no presystolic accentuation due to AF and severe MS. Opening snap (OS) with irregular timing due to AF was still heard in this patient, although the sound was less audible because of the loss of atrial contraction in AF. There was an OS that was occurred earlier after the aortic component of the second heart sound (A2) in this patient with severe MS, so that there was a short A2–OS interval. Meanwhile, lung auscultation showed clear vesicular lung sounds (Tables 1, 2).

**Table 2:** Timeline of onset and diagnostic and therapeutic management in this patient with acute limb ischemia (ALI) Rutherford IIa.

Diagnostic/therapeutic procedures	Onset (day 0)	Day 1	Day 2	Day 3	Day 4	Day 9	Day 12
Onset (symptoms and signs)	+						
Non-invasive Diagnostic procedure (ECG, chest X-ray, echocardiography)	+						
Duplex ultrasound	+						
Unfractionated heparin	+	+	+	+	+	+	
First PMT (thromboaspiration)	+						
Warfarin						+	+
ICCU stabilization and blood transfusion	+	+	+	+			
CT angiography		+					
Second PMT (balloon)					+		
Discharge							+

**Figure 1:**
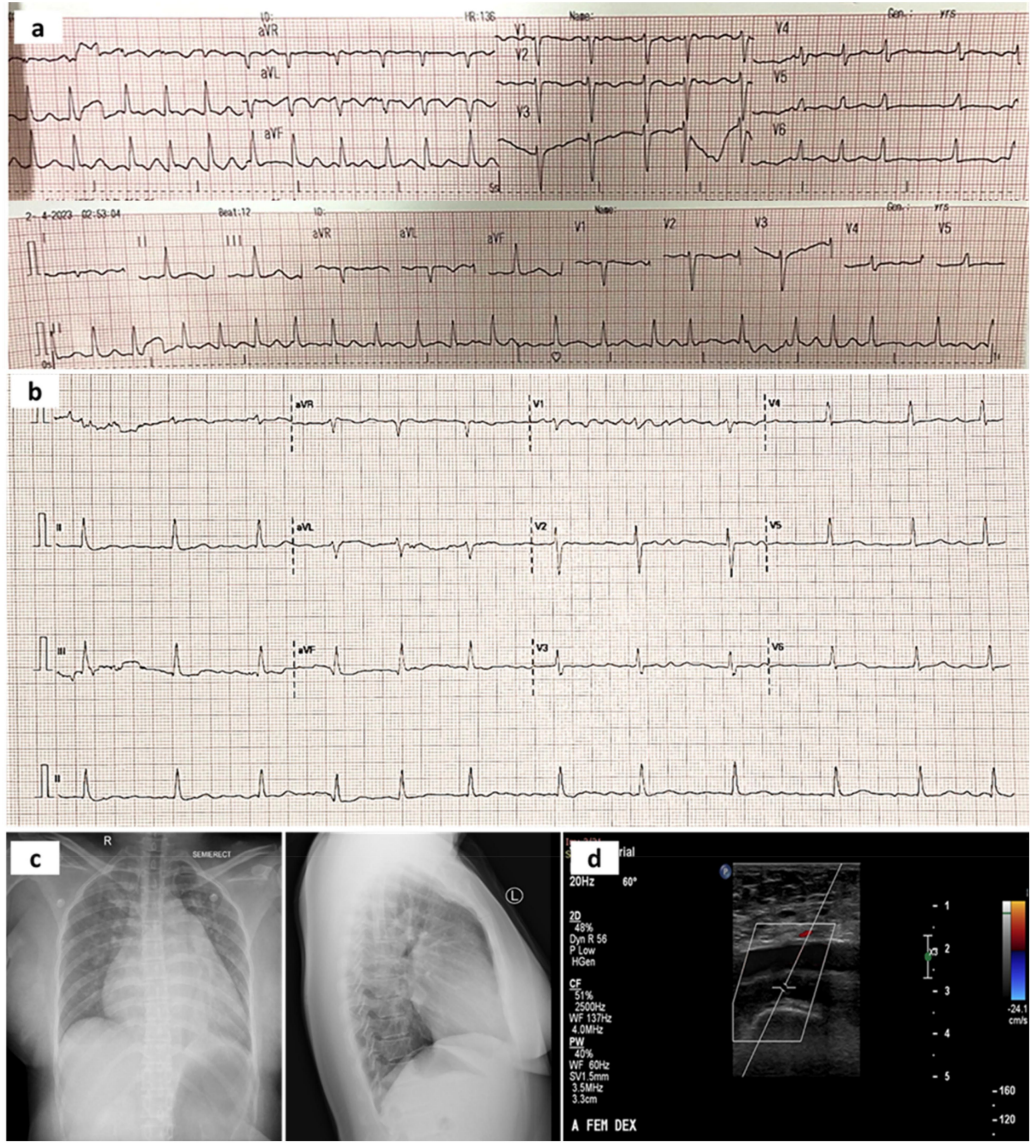
An electrocardiogram shows atrial fibrillation (AF) with rapid ventricular response on admission **(a)** and AF with normal ventricular response after rate control management **(b)**. A chest X-ray shows an enlarged left atrium, right atrium, and right ventricle **(c)**. A duplex ultrasound shows no blood flow from the femoral to the popliteal and tibial arteries on both sides **(d)**.

An electrocardiogram (ECG) showed AF with a rapid ventricular response rate of 130–140 beats per minute on admission (Figure 1a) and AF with a normal ventricular rate response rate of 70–80 bpm after rate control management of digoxin injection (Figure 1b). A chest X-ray showed an enlargement of the left atrium (LA), right atrium (RA), and right ventricle (RV) (Figure 1c, Table 2). Laboratory tests revealed leucocytosis.

A bedside Doppler ultrasound (DUS) of both legs showed no blood flow from the femoral to the popliteal and tibial arteries on both sides (Figure 1d) (Table 2). We were unable to perform emergency CT angiography (CTA) at this point of time; however, we opined that CTA remained the gold standard and current imaging guidance in ALI for defining the precise extent of the thrombus, the inflow/outflow anatomy, and access in which was also particularly crucial in suspected aorto-iliac saddle embolus and was also essential for safe therapeutic planning. As the patient had already shown clear clinical symptoms and signs of ALI, he was planned for immediate invasive angiographic therapy. The clinician also expressed concern that CTA might delay the timing for the immediate invasive angiography procedure, in which a quick CTA result was sometimes not found feasible in our health center at that point of time.

**Figure 2:**
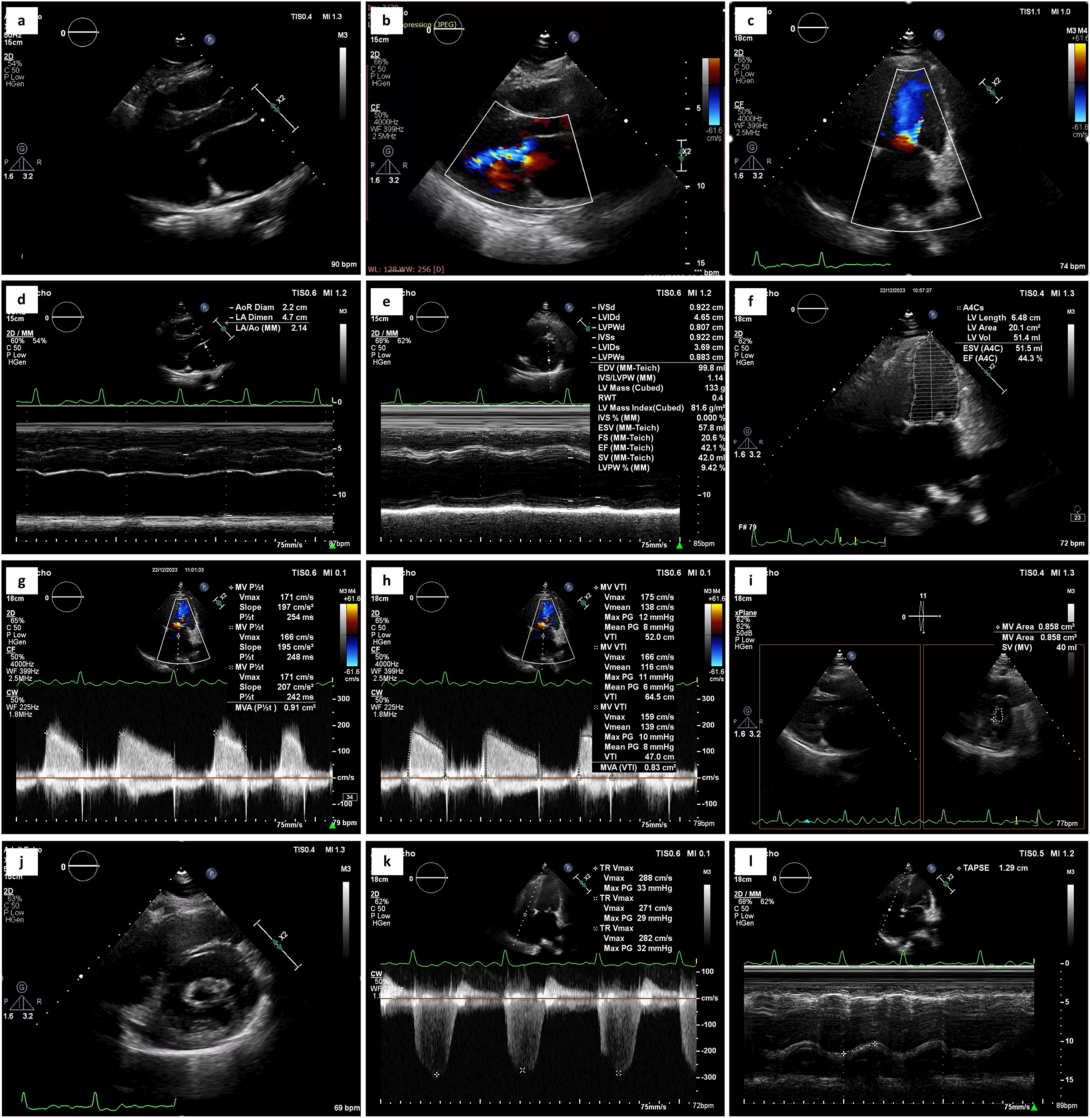
Transthoracic echocardiography of mitral stenosis shows a hockey-stick appearance of the anterior mitral leaflet in the 2D PLAX view **(a)**. A color Doppler shows a stenotic jet of mitral stenosis in the PLAX view **(b)** and the four-chamber view **(c)**. Transthoracic echocardiography shows an enlarged left atrium **(d)**. Reduced left ventricular systolic function seen after the use of the Teichholz **(e)** and biplane methods **(f)**. Measurement of the mitral valve area (MVA) by the pressure half time (P1/2 T) **(g)**, by continuity equation using velocity-time integral (VTI) **(h)**, and by planimetry methods **(i)**. An anatomical valve morphology of mitral stenosis in the PSAX view at the level of the mitral valve **(j)**. The shape and density of the CW Doppler waveform in tricuspid regurgitation in the four-chamber view **(k)**. Measurement of the right ventricular (RV) function shows reduced tricuspid annular plane systolic excursion (TAPSE) **(l)**.

A bedside transthoracic echocardiogram (TTE) indicated severe mitral stenosis due to rheumatic mechanism after the valve morphology was assessed, as testified by leaflet thickening, commissural fusion, and the presence of subvalvular disease (Figures 2a–l). LA, RA, and RV dilatation were revealed (Figures 2a–d,f). The mitral valve area (MVA) measured 0.91 cm^2^ by P1/2 T, 0.83 cm^2^ by Continuity Equation using velocity-time integral (VTI), and 0.85 cm^2^ by planimetry methods (Figures 2g–i). The Wilkins score of 8 for valve morphology showed a reduced mobility midportion and base of leaflets (leaflet mobility score 2), a midleaflet and margin valve thickening (leaflet thickening score 2), a thickening of chordae up to one-third of chordal length (subvalvular apparatus thickening score 2), and a scattered area of valvular calcification that was confined to the leaflet margins (valvular calcification score 2) (Figure 2j). The TTE also showed that the mean and peak pressure gradients across the mitral valve were 8 and 11 mmHg, consecutively (Figure 2h). A moderate tricuspid regurgitation was revealed by Continuous Wave Doppler in the four-chamber view (Figure 2k). No LA thrombus or dense spontaneous echo contrast was indicated by TTE (Figures 2a,b), and a reduced RV function was revealed by TAPSE (TAPSE 13 mm) (Figure 2l) (Table 2). A transesophageal echocardiogram (TOE) was strongly recommended according to the guidelines to rule out left atrial appendage thrombus; however, we could not perform a TOE because we did not find it feasible in that clinically urgent situation in which TTE had already provided sufficient information before planned revascularization. Because the TTE had already confirmed severe rheumatic mitral stenosis and significant chamber dilatation as well as atrial fibrillation in ECG, there was already sufficient clinical evidence to support the suspected embolic source that was presumed cardioembolic.

The patient was then diagnosed with bilateral ALI Rutherford IIa caused by systemic embolism, severe MS, AF, and dyslipidemia. The patient was administered a bolus of 4,000 units of unfractionated heparin (UFH), followed by a maintenance dose of 750 units per hour, targeting an Activated Partial Thromboplastin Time (APTT) of 2.0–3.0. We also administered morphine via a syringe pump as an analgesic. In addition, the patient received oral medications such as aspirin, clopidogrel, atorvastatin 40 mg, and digoxin for rate control. The use of beta blockers was postponed at admission as the patient was experiencing pulmonary congestion and rales at the lung bases, and therefore, digoxin was preferred at that time for rate control (digoxin tab 0.25 mg once daily). Bicarbonate, folic acid, N-acetylcysteine, allopurinol, and vitamin C were also administered as adjunctive treatment to prevent reperfusion injury. We scheduled the patient for an urgent arteriography and an emergency revascularization by percutaneous mechanical thrombectomy (PMT).

We performed arteriography via single right femoral artery access using a Judkins Right (JR) 3.5/6F guiding catheter, which revealed a total occlusion at the distal abdominal aorta at the aortoiliac bifurcation, indicating a thrombus-type occlusion. Because of the lack of specialized PMT devices (such as AngioJet and Rotarex), we modified the PMT using a vacuum-assisted wall suction, positioning the vacuum as close to the patient as possible. We performed the PMT with the thromboaspiration method starting from the distal abdominal aorta to the left iliac and left femoral arteries. The vascular access size or sheath size was 6 French (6Fr) femoral access. The JR 3.5/6F guiding catheter was initially placed at the distal thrombus. We administered anticoagulation during the thromboaspiration procedure with UFH with an activated clotting time of 250–300 s. The thromboaspiration device was passed through the wire and was then placed at the distal thrombus. It was connected to the wall-suction system and the aspiration process was started from the distal thrombus; the device was moved toward its proximal position up to the midabdominal aorta, and was further pulled to the left iliac and left femoral arteries. During aspiration, the maximum vacuum pressure or maximum suction pressure was approximately 80–100 mmHg. The number of times that the thromboaspiration device passed through the lesion and performed aspiration processes was approximately five times that of aspiration with a duration of 15–20 s per aspiration. The device would aspirate a volume of blood of 700 cc. We did not use distal embolic protection. This procedure showed a fluoroscopy time of 50 min with a radiation dose or dose area product of 120 Gy·cm^2^ and a consumed contrast volume of 100 cc.

**Figure 3:**
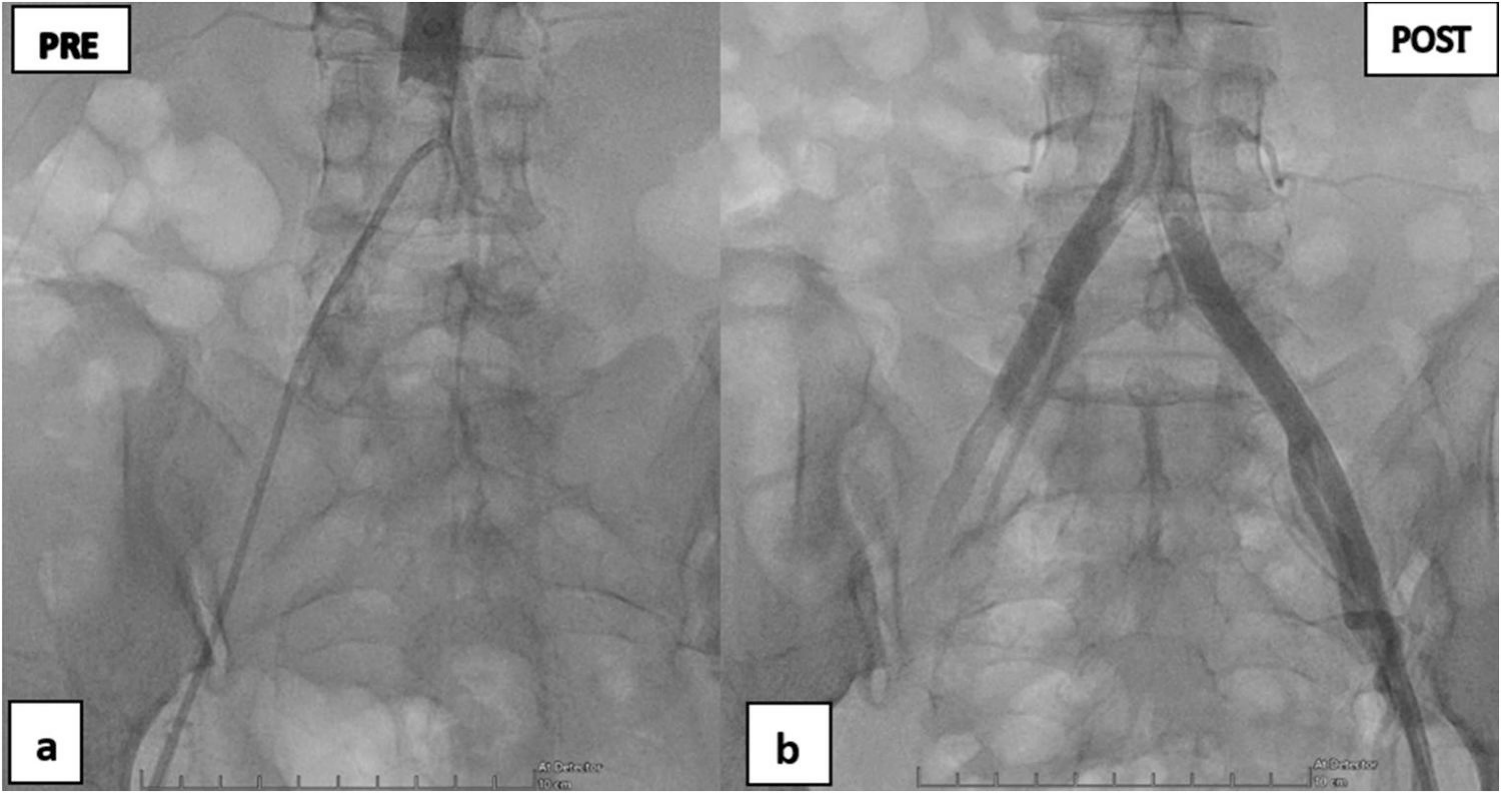
Arteriography prerevascularization or prepercutaneous mechanical thrombectomy (PMT) via the right femoral artery access using a JR 3.5/6F guiding catheter shows total occlusion in the distal abdominal aorta at the aortoiliac bifurcation **(a)**. An evaluation after vacuum-assisted thromboaspiration that was performed using conventional wall suction shows that the contrast could flow to the distal left lower extremity, but there an occlusion remains in the right femoral artery **(b)**.

The final angiographic result after the first thromboaspiration procedure was a TIMI-like II flow at the distal right femoral artery with good distal runoff and a TIMI-like III flow until the distal left lower extremity with good distal runoff. The evaluation results showed that the contrast could flow to the distal left lower extremity, but an occlusion remained in the right femoral artery (Figures 3a,b). Because of the significant blood loss (almost 900 cc) and a drop in hemoglobin >2 g/dL (from 14.5 to 9.4 g/dL) that was taken from venous blood sampling, we decided to defer the procedure on the right leg, focusing on stabilizing the patient's condition and performing a transfusion first in the intensive cardiovascular care unit (ICCU). This drop in hemoglobin levels was classified as a “procedural bleeding complication.” There were mild hemodynamic effects such as tachycardia but not hypotension. The patient received a transfusion of 2 units of Packed Red Cell (PRC). There was no hematoma in the access site nor retroperitoneal bleeding.

In the ICCU, we performed a multi-slice computed tomography (MSCT) angiography on both the right and left legs of the patient. The results showed that in the arterial phase, an occlusion and thrombus in the distal right femoral artery (approximately 11.22 cm) remained. We also conducted a DUS evaluation in the ICCU, which showed that there was flow in the left leg with a triphasic wave reaching the anterior and posterior tibial arteries. However, there was still no flow in the right profunda femoral artery and below the popliteal artery.

**Figure 4:**
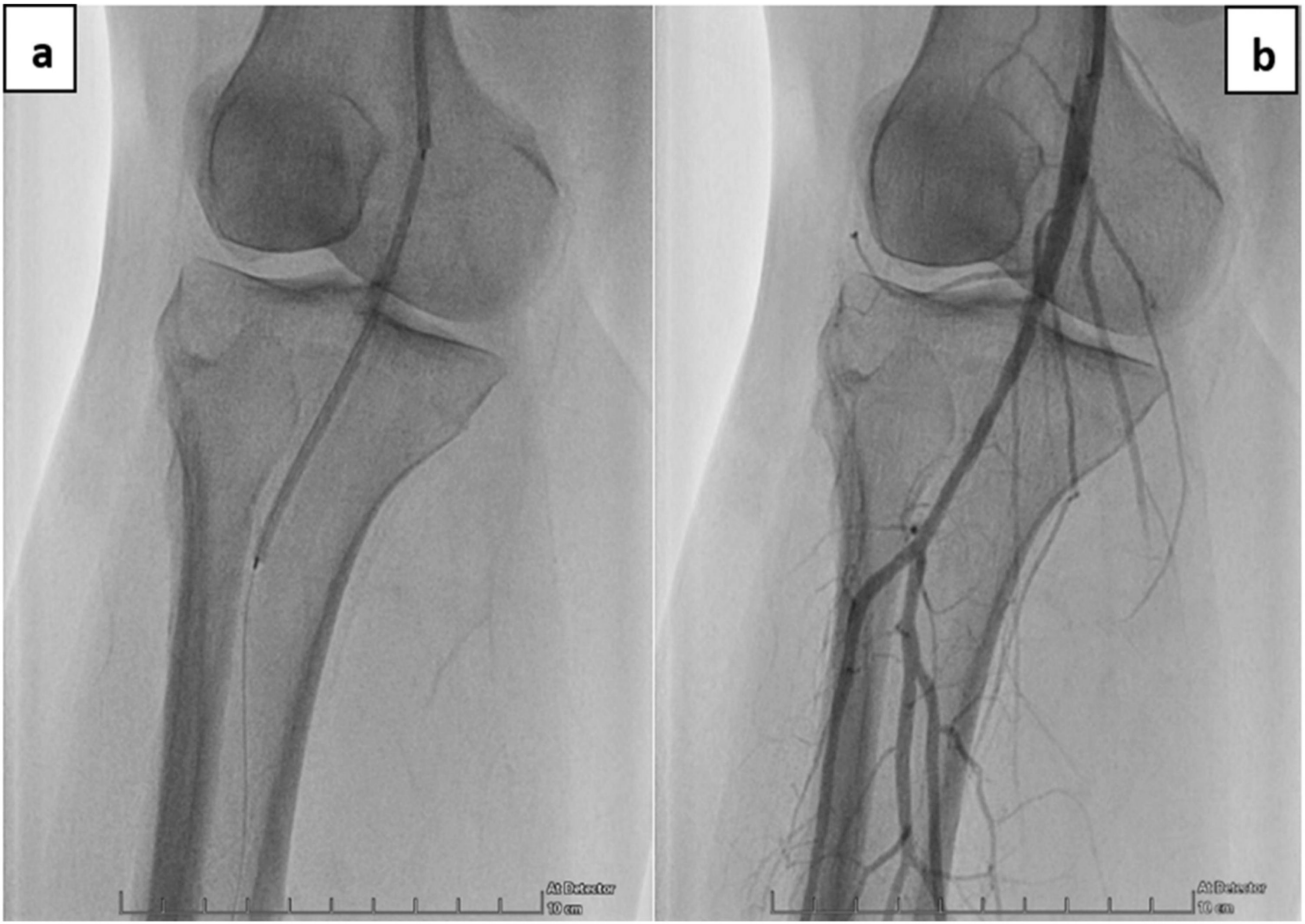
A second PMT was performed on the right leg through the left femoral artery using a JR 3.5/6F guiding catheter. We performed dilation with a Coyote balloon 3.0 × 80 mm at 10–15 atm **(a)**. An angiographic evaluation shows good flow to the distal arteries **(b)**.

After the hemoglobin level increased to 11.0 g/dL and the patient's hemodynamic status was stabilized with no active bleeding, a second PMT was performed on the right leg at a different point of time during the procedure. Access was obtained through the left femoral artery using a JR 3.5/6F guiding catheter. The bifurcation of the aortoiliac arteries facilitated good flow. However, consistent with the MSCT results, a thrombus occlusion remained in the distal right femoral artery up to the proximal right popliteal artery. We crossed the occlusion using a Sion Blue wire, followed by a percutaneous transluminal balloon angioplasty that was necessitated due to stenosis in the right popliteal–tibial artery. We performed dilation with a Coyote balloon 3.0 × 80 mm at 10–15 atm (Figure 4a). An angiographic evaluation showed good flow to the distal arteries (Figure 4b).

**Figure 5:**
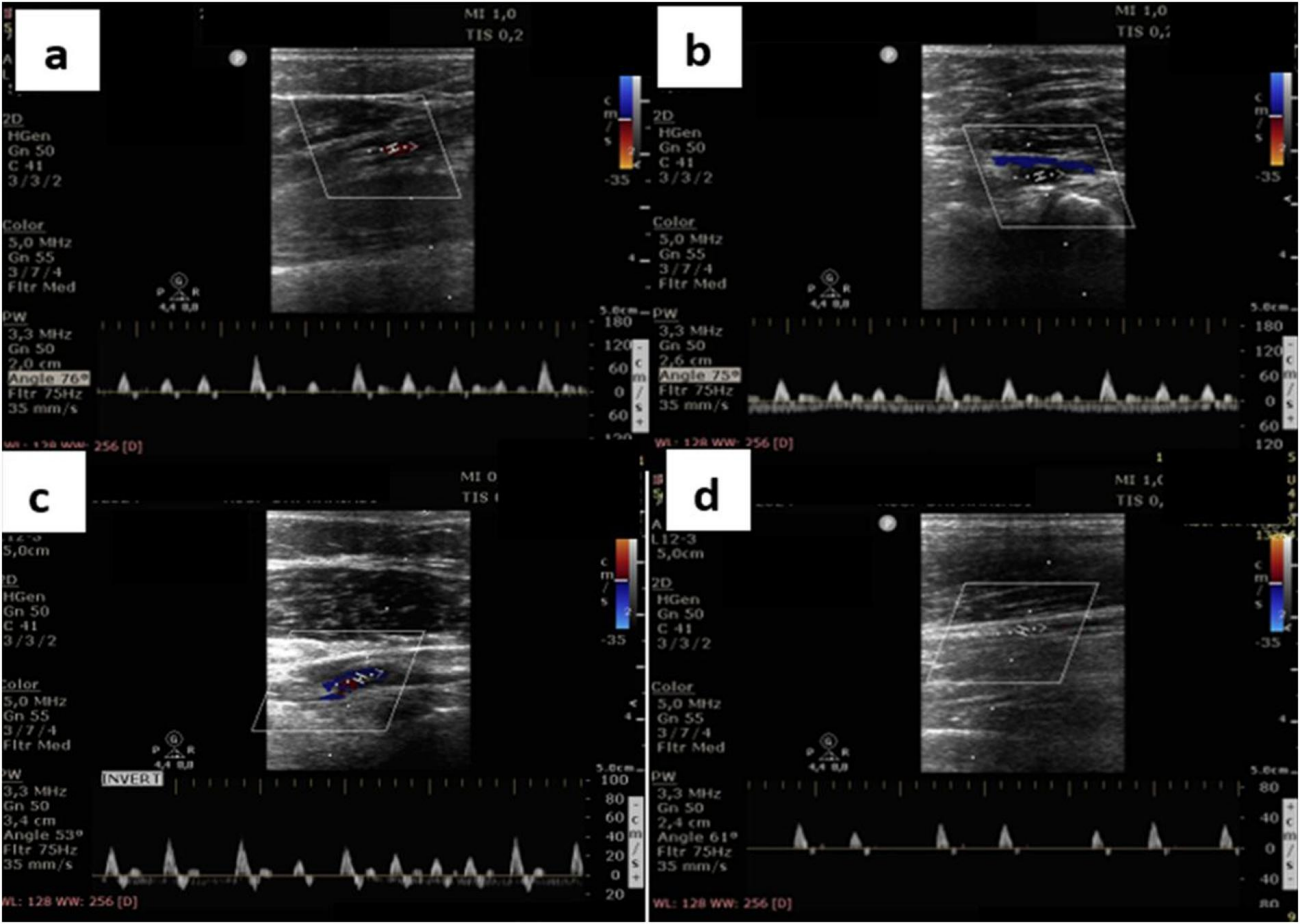
A duplex ultrasound evaluation of the left femoral arteries **(a)**, left tibial arteries **(b)**, right femoral arteries **(c),** and right tibial arteries **(d)** shows normal flow with triphasic morphology at day 1 after the second procedure.

A clinical evaluation post-PMT revealed that the patient reported no pain in the legs, with no numbness or tingling. The extremities were warm and not pale, the dorsalis pedis pulse was strong, and peripheral oxygen saturation was normal. A duplex ultrasound evaluation of both the right and left legs showed normal flow from the femoral, popliteal, to the tibial arteries, with triphasic morphology (Figures 5a,b) (Table 3). During hospitalization (after revascularization), the patient received dual antiplatelet therapy (DAPT) that was a combination of aspirin and clopidogrel, a P2Y12 inhibitor, as well as unfractionated heparin. This unfractionated heparin was bridging the vitamin K antagonist (VKAs) warfarin before discharge until a therapeutic International Normalized Ratio (INR) of 2.0–3.0 was achieved. The total length of hospitalization was 12 days.

**Table 3:** Symptoms, signs, and Doppler examination in pre- and postrevascularization procedures in the patient case with acute limb ischemia (ALI) Rutherford IIa.

Findings	Complaints	Pulselessness	Pallor	Poikilothermic	Capillary refill of lower extremities	Sensory changes	Reduced motoric strength: paralysis	Doppler signals: artery	Doppler signals: vein
Prerevascularization procedure	Severe pain in both lower extremities	No pulse at both lower extremities	Pale with mottled discoloration on the soles	Cold to the touch	Slower (right >2 s/left >2 s)	Partial. Numbness and tingling in the legs	None	Inaudible	Audible
Postrevascularization procedure	No pain	Normal pulse	Normal color	Normal	Normal (right <2 s/left <2 s)	Normal	None	Audible	Audible

At discharge, the patient was given aspirin (for 1 week) and a long-term VKA, namely, warfarin, with a target (INR) of 2.0–3.0. As this patient had rheumatic MS, VKAs remained indicated as the first-choice anticoagulation. At discharge, the hemoglobin level was 10.6 g/dL and the creatinine level was 0.75 mg/dL. During the follow-up visit, the patient reported good clinical results and showed full recovery. One year later, the patient underwent mitral valve replacement (MVR) with good outcomes and no complications. A TOE was performed before MVR.

## Discussion

An acute aortic occlusion is a rare but life-threatening medical condition that may result from an ASE, thrombosis of an atherosclerotic aorta, or aortic dissection. ASE is an uncommon vascular disorder that develops when a blood clot, typically originating from the proximal arteries, most often from the left side of the heart, travels through the arterial system and becomes lodged at the bifurcation of the aorta. This critical location of obstruction disrupts the normal flow of blood to the organs and tissues downstream, particularly affecting both lower extremities (6). Although this condition is rare, it is clinically significant, accounting for roughly 10% of all cases of peripheral arterial embolism. The most frequent sources of these emboli are thrombi that form in the left atrium, especially in patients with atrial fibrillation (7). The abrupt blockage at the aortic bifurcation can result in severe hemodynamic and metabolic consequences, and if not promptly recognized and treated, may lead to limb-threatening ischemia, organ dysfunction, or even death (8).

In the case of the patient in this study, the most probable cause of ASE resulting in the total occlusion of the abdominal aorta was related to atrial fibrillation and her underlying mitral stenosis due to rheumatic heart disease, which led to the formation of thromboembolism. This was in line with a report by Ding et al. that retrospectively reviewed patients with ASE and their demographic, clinical, ancillary testing, treatment, and outcome data. They showed that the most commonly associated cardiac diseases in ASE were AF or atrial flutter (89%); rheumatic heart disease, valvular heart disease, or both (72%); and congestive heart failure (56%) (2).

In our patient, the chief complaint was resting pain and partial sensory loss. This was in line with a report by Ding et al. that showed that resting pain was present in all patients, and sensory or motor deficits were present in 67% of patients. Ding et al. reported that 17 patients (94%) presented with aortic embolism below the renal arteries (2).

Ding et al. found that, although ASE was uncommon, it was associated with high morbidity and mortality. They also reported that 83% of patients underwent a bilateral transfemoral embolectomy, and 17% of patients received no intervention. The overall mortality rate was 33%, with a postprocedural mortality rate of 20%. Major morbidity occurred in 60% of patients. Six lower extremities were amputated in 4 patients, and acute renal failure developed in four patients. The incidence of postembolectomy internal iliac artery embolism was reported in 58% of patients, and pelvic ischemia developed in one young patient (2).

According to the ALI algorithm from the European Society of Vascular Surgery (ESVS) 2020, the primary goal of ALI management is to restore blood flow to an adequate level. Upon an initial diagnosis of ALI, a bolus of heparin 70–100 units/kg is administered, followed by maintenance, oxygen supplementation, and pain management. In our case, for the initial treatment, the patient received a bolus of 4,000 units of heparin, followed by a maintenance dose of 750 units per hour, targeting an APTT of 2.0–3.0, and morphine was administered via a syringe pump as an analgesic. Subsequently, imaging was used to evaluate the Rutherford class of the patient, determining whether the condition is still viable, threatened, or irreversible (2, 4). For patients classified as Rutherford class IIa or marginally threatened, as we found in our case, revascularization should be performed as quickly as possible using endovascular and surgical methods or a combination of both (9). Ding et al. also recommended CT angiography for anatomical diagnosis (2); unfortunately, however, we did not find it feasible to perform a quick CT angiography in our patient at that point of time.

Revascularization strategies in patients with ASE are critical to limb salvage and survival, given the high morbidity and mortality associated with this rare vascular emergency. Ding et al. reported that bilateral transfemoral embolectomy was also the treatment of choice (2). PMT is a rapid and effective method to re-establish blood flow, in which specialized catheters are used to aspirate or fragment thrombi at the aortic bifurcation (10). Vacuum-assisted thromboaspiration has emerged as a promising alternative percutaneous technique in the management of ALI, offering a minimally invasive alternative to traditional open surgical thrombectomy and catheter-directed thrombolysis. This method employs continuous vacuum suction to effectively remove thrombotic occlusions from the peripheral arteries, thereby rapidly restoring blood flow (11). In our patient, due to the lack of a more sophisticated system, such as the Penumbra/Indigo® aspiration device, we used a conventional wall suction instead. As we were still employing conventional wall suction, the bleeding during the initial procedure was significant. Consequently, we decided to defer the remaining procedure to another day, allowing the patient's condition to stabilize and blood transfusions to be administered beforehand. Previous studies have demonstrated high technical success rates and favorable clinical outcomes, with limb salvage rates exceeding 90% and low perioperative complication rates ranging from 2% to 14%. Collectively, current evidence supports vacuum-assisted thromboaspiration as a safe, effective, and efficient endovascular option for patients presenting with ALI, with growing acceptance in vascular surgery and interventional radiology practice (12–14).

As the adapted wall-suction technique has led to a blood loss of approximately 900 cc and a precipitous drop in hemoglobin levels (from 14.5 to 9.4 g/dL), it may expose the patient to a significant iatrogenic exsanguination risk. Although potentially life-saving in a resource-depleted environment, this method may carry a substantially higher risk of hemodynamic instability compared with regulated, closed-loop aspiration systems. This complication has forced a staged intervention in our patient, resulting in a delay of several days for the final revascularization of the right lower extremity in a time frame that demanded a more rigorous discussion regarding the risks of neuromuscular necrosis and systemic inflammatory response (1–5, 9).

Rutherford IIa limbs are classified as “marginally threatened” ALI, which are defined as minimal sensory loss (typically confined to the toes), intact motor function, and absent arterial Doppler signals but audible venous signals. The limbs are considered salvageable if treated promptly. However, allowing a Rutherford IIa limb to remain untreated for several days might fundamentally change the pathophysiology. As Rutherford IIa (“marginally threatened”) limbs were left untreated for several days in our patient, she was found to be no longer dealing with early ischemia alone but would have experienced progressive ischemia with evolving tissue necrosis, profound metabolic derangement, and a substantially increased risk of severe reperfusion injury if revascularization had been eventually attempted. It was characterized by Adenosine Triphosphate (ATP) store depletion, rapid transition from the cell's oxidative phosphorylation to anaerobic glycolysis (anaerobic metabolism), intracellular acidosis, ion pump failure (particularly Na⁺/K⁺-ATPase), muscle necrosis, and accumulation of lactate, hydrogen ions, potassium, myoglobin, purines (hypoxanthine), reactive oxygen species precursors, and other toxic metabolites. As ATP levels fall, it may cause cellular ion dysregulation such as intracellular sodium accumulation, intracellular water accumulation following sodium accumulation that was causing cellular swelling, and increased calcium influx due to membrane pump failure. The elevated intracellular calcium may activate proteases, phospholipases, and endonucleases. These processes may accelerate irreversible muscle injury and membrane destruction. Meanwhile, revascularization at that stage might have possibly carried a substantial risk of reperfusion injury, including severe hyperkalemia, metabolic acidosis, rhabdomyolysis, acute kidney injury, compartment syndrome, oxidative stress, and systemic inflammatory responses. Consequently, management after such a delay requires a careful evaluation of both limb viability and the patient's systemic risk, because restoring perfusion might itself precipitate life-threatening complications (1–5, 9, 15).

We did not pursue an immediate surgical embolectomy once the percutaneous approach was aborted because of acute anemia, although there would be a possible risk of permanent functional loss in prolonged ALI as we needed to ensure whether this acute anemia might have signaled an unresolved bleeding source. Our utmost concern was that acute anemia was often not merely a laboratory abnormality—it might indicate active hemorrhage. This particular acute anemia due to active hemorrhage or other possible situations may lead to a physiologically unstable status for surgery in specific patients. In our patient, we occasionally had to choose between maximizing limb salvage and minimizing mortality risk. If a patient develops significant anemia plus significant comorbid instability, such as heart failure due to mitral stenosis and atrial fibrillation, as in our patient, the cardiovascular team may conclude that immediate surgery poses unacceptable systemic risk. In that setting, preserving life should take precedence over preserving the limb (9, 15).

An antithrombotic strategy is also important in patients with ALI as in our patient. The patient was administered with DAPT consisting of aspirin and clopidogrel soon before and after emergency revascularization (PMT). The 2024 European Society of Cardiology (ESC) guidelines for the management of peripheral arterial and aortic diseases state that DAPT or rivaroxaban (2.5 mg b.i.d.) and aspirin (100 mg o.d.) should be considered in patients with ALI following revascularization if they are not on anticoagulation for other reasons (15). Moreover, a study by Tepe et al. also showed that DAPT reduced peri-interventional platelet activation and improved functional outcome without higher bleeding complications. The degree of platelet activation was assessed by the concentration of *ß*-thromboglobulin (*ß*-TG) and human-soluble CD40 ligand (CD40L) as both are specific markers for platelet activity and chronic inflammatory processes such as atherosclerosis. They showed that an individual-tailored DAPT seemed desirable for endovascularly treated patients with Peripheral Arterial Disease (PAD) (16). In the case of our patient, during hospitalization (after revascularization), she received DAPT that was a combination of aspirin and clopidogrel, a P2Y12 inhibitor, as well as heparin. At discharge, the patient was given aspirin and a long-term plan of VKA, namely, warfarin. As this patient had rheumatic MS, VKAs remained indicated as the first-choice anticoagulation. The MVR was performed 1 year later because of institutional preference. Moreover, the cardiovascular team decided to perform MVR because the patient had experienced clinically significant cardiac emboli resulting in ALI-ASE.

## Conclusion

Early diagnosis of ASE and comprehensive, rapid management could reduce the risk of irreversible tissue death or even multiorgan damage. Percutaneous mechanical thrombectomy could serve as a quick and applicable revascularization modality for ASE or ALI. Modified wall-suction thromboaspiration may represent a feasible bailout or resource-adapted option when dedicated thrombectomy devices are unavailable, but its safety, blood loss risk, and reproducibility require further evaluation.

## Data Availability

The datasets generated and/or analysed during the current study are not publicly available due to patient privacy and confidentiality considerations and institutional ethical restrictions. De-identified data supporting the findings of this study are available from the corresponding author upon reasonable request and subject to approval by the relevant ethics committee and institution.
